# Effects of Methionine Supplementation on the Expression of Protein Deposition-Related Genes in Acute Heat Stress-Exposed Broilers

**DOI:** 10.1371/journal.pone.0115821

**Published:** 2015-02-25

**Authors:** Ana Paula Del Vesco, Eliane Gasparino, Daiane Oliveira Grieser, Vittor Zancanela, Débora Marques Voltolini, Angélica Souza Khatlab, Simone Eliza Facioni Guimarães, Maria Amélia Menck Soares, Adhemar Rodrigues Oliveira Neto

**Affiliations:** 1 Department of Animal Science, Universidade Estadual de Maringá—UEM—Maringá, Paraná, Brazil; 2 Department of Animal Science, Universidade Federal de Viçosa—UFV—Viçosa, Minas Gerais, Brazil; 3 Department of Genetics, Universidade Federal Rural do Rio de Janeiro—UFRRJ—Seropédica, Rio de Janeiro, Brazil; 4 Evonik-Degussa, São Paulo, São Paulo, Brazil; Universidad Nacional Autonoma de Mexico, MEXICO

## Abstract

The objective of this study was to evaluate the effect of heat stress and methionine supplementation on the gene expression of insulin-like growth factor I (IGF-I), growth hormone receptor (GHR), phosphatidylinositol 3-kinase, and regulatory 1 (PI3KR1) in the liver, as well as the expression of the atrogin 1 and cathepsin L2 (CTSL2) genes in the breast muscle of broilers. Broilers from 1–21 and 22–42 days of age were divided into three treatments related to methionine supplementation as follows: without methionine supplementation (MD), recommended level of methionine (DL1), and excess supplementation of methionine (DL2). The animals were either maintained at a thermal comfort temperature or exposed to heat stress (HS) (38°C for 24 hours, starting on day 20 or day 41 for experiments 1 and 2, respectively). The heat stress increased the body temperature at both ages. Starter period: The HS animals presented increased plasma creatinine content (P<0.0001) and the highest CTSL2 gene expression (P<0.0001). The methionine supplementation increased the IGF-I (P = 0.0144) and GHR (P = 0.0011) gene expression and decreased the CTSL2 (P = 0.0004) and atrogin 1 (P = 0.0012) gene expression. Grower period: Significant effects for the interaction between supplementation and environment were observed for GHR (P = 0.0252) and CTSL2 (P = 0.0011) gene expression. The highest GHR expression was observed in animals that remained in thermal comfort on the DL2 diet, and the lowest expression occurred in the HS animals fed the MD diet. For CTSL2, the HS animals fed the MD diet presented the highest CTSL2 gene expression, and the lowest expression was observed in the animals maintained at thermal comfort on DL1 and DL2 diets. Only methionine supplementation had effect on atrogin-1 gene expression (P<0.0001), with higher methionine content in the diet lower atrogin-1 gene expression was observed. Our results suggest that heat stress induces greater protein degradation and that methionine supplementation could induce protein deposition because methionine increased the expression of genes related to protein synthesis and decreased the expression of genes related to protein breakdown.

## Introduction

Growth occurs because of protein deposition based on a balance between protein synthesis and breakdown. These two distinct pathways are products of the same biological route [1], and hormone concentration, diet, and the environment are factors that can determine which of these two pathways will prevail.

Methionine supplementation has a positive effect on protein synthesis by acting on several factors, and methionine supplementation affects the expression of genes related to growth. Higher mRNA expression in the liver [2] and increased levels of circulating IGF-I are often associated with animals on supplemented diets [3]. Methionine has also been reported to be an inhibitor of enzymes, such as atrogin 1, which participate in degradation via the ubiquitin-proteasome pathway [4].

Moreover, similar to when there is a lack of nutrients, such as amino acids, high temperatures may also be blamed for promoting proteolysis. In addition to the effects of increased ROS production on metabolism, stress is also associated with lower IGF-I expression, increased expression of components of the ubiquitin-proteasome pathway [5], activation of transcription factor Forkhead box (FoxO) signaling [6], and autophagy induction [7].

This study evaluated the hypothesis that heat stress (HS) can stimulate the body to undergo higher levels of proteolysis and that methionine supplementation may contribute not only to less degradation but also to higher protein synthesis, thereby reducing the oxidative damage caused by HS. Therefore, we evaluated the effect of HS and methionine supplementation on the gene expression of insulin-like growth factor I (IGF-I), growth hormone receptor (GHR), and phosphatidylinositol 3-kinase, regulatory 1 (PI3KR1) in the liver, and expression of the atrogin 1 and cathepsin L2 (CTSL2) genes in the breast muscle of broilers from 1–21 and 22–42 days of age. The genes encoding the CTSL2 and atrogin 1 enzymes were selected for analysis in this study because these enzymes function in the lysosomal degradation route and the pathway of the ubiquitin-proteasome complex, respectively. For PI3KR1, this enzyme was selected because it participates in the cascade of metabolic reactions activated by IGF-I.

## Materials and Methods

The Committee on Animal Care of the Universidade Estadual de Maringá—Brazil approved this study.

### Experimental Design and Animals

**Experiment 1-Starter period-1–21 days old**. A total of 180 male broilers (Cobb 500) *(Gallus gallus)* were used for the starter period experiment. The animals were divided into three treatments related to methionine supplementation as follows: without supplementation (MD, n = 60), supplementation with the recommended level of methionine (DL1, n = 60) [8], and excess supplementation with methionine (DL2, n = 60) (Table 1). The animals were distributed in a completely randomized design with four replications (pens) per treatment, and each replicate consisted of 15 birds. Throughout the experimental period, the animals had free access to food and water.

**Table 1:** Experimental diets, centesimal composition (expressed as-fed basis).

	Starter period			Grower period		
Ingredients	MD^1^	DL1	DL2	MD	DL1	DL2
Corn 7.8% CP	550.75	548.80	542.70	600.00	598.20	592.05
Soy bean meal 46.0% CP	373.00	373.00	374.00	324.00	324.00	325.00
Soy oil	39.00	38.00	36.00	46.00	45.00	43.00
Salt	4.50	4.50	4.50	4.30	4.30	4.30
Calcareous 38%	11.60	11.60	11.60	9.30	9.30	9.25
Dicalcium phosphate 20%	15.25	15.25	15.30	10.65	10.70	10.70
DL- Methionine 99%	-	2.95	10.00	-	2.75	10.00
L- Lysine HCl 78%	1.55	1.55	1.55	1.55	1.55	1.50
L-Threonine 78%	0.35	0.35	0.35	0.20	0.20	0.20
Premix^2^	4.00	4.00	4.00	4.00	4.00	4.00
Total	1000.00	1000.00	1000.00	1000.00	1000.00	1000.00
Composition analysis (%)						
CP	21.61	21.77	22.191	19.73	19.88	20.36
Lysine digestible	1.19	1.19	1.20	1.08	1.08	1.08
Met+Cis digestible	0.58	0.88	1.57	0.54	0.81	1.53
Threonine digestible	0.78	0.78	0.78	0.70	0.70	0.70
Tryptophan digestible	0.24	0.24	0.24	0.22	0.22	0.22
Valine digestible	0.92	0.92	0.92	0.84	0.84	0.84
Isoleucine digestible	0.86	0.86	0.86	0.77	0.77	0.77
Arginine digestible	1.38	1.38	1.38	1.24	1.24	1.24
Composition Calculated (%)^3^						
Ca	0.88	0.88	0.88	0.68	0.68	0.68
P	0.45	0.45	0.45	0.35	0.35	0.35
Na	0.20	0.20	0.20	0.19	0.19	0.19
AME (kcal/kg)	3052.51	3051.94	3051.38	3169.60	3168.53	3168.60

^1^MD, methionine deficient; DL1, recommended level of methionine supplementation; DL2, excess methionine supplementation.

^2^Supplied by kilogram of diet: retinyl-acetate, 3.44 mg; cholecalciferol, 50 μg; DL-*α-*tocopherol, 15 mg; thiamine, 1.63 mg; riboflavin, 4.9 mg; pyridoxine, 3.26 mg; cyanocobalamin, 12 μg; D-pantothenic acid, 9.8 mg; D-biotin, 0.1 mg; menadione, 2.4 mg; folic acid, 0.82 mg; niacinamide, 35 mg; selenium, 0.2 mg; iron, 35 mg; copper, 8 mg; manganese, 60 mg; zinc, 50 mg; I, 1 mg; choline, 650 mg; salinomycin, 60 mg; avilamycin, 5 mg; butyl hydroxy toluene, 80 mg.

^3^Feed formulations were based on the total amino acids of corn and soybean meal as analyzed by Evonik Degussa (Hanau, Germany). The digestibility coefficient suggested by Rostagno et al. (2011) was used to obtain the digestible amino acids. AME: apparent metabolizable energy. The amino acids, crude protein and dry matter were analyzed by Evonik Degussa (Hanau, Germany).

The 180 birds distributed among the different treatments were raised in a chamber at thermal comfort until 20 days of age, when 90 animals (30 from each diet) were subjected to acute heat stress of 38°C for 24 hours. During the stress period, the remaining 90 animals (30 from each diet) were removed from the chamber and housed in a thermoneutral environment throughout the period. After 24 hours of stress, the animals from both groups (comfort and heat stress, HS) were slaughtered via cervical dislocation at 21 days.

**Experiment 2-Grower period-22–42 days old**. A total of 180 male broilers (Cobb 500) (*Gallus gallus*) were used for the grower period experiment. The animals were raised conventionally until 21 days of age and were fed a balanced diet to meet their nutritional demands [8]. After 21 days, the animals were divided into groups similar to experiment 1.

The 180 birds distributed among treatments were raised in a climatized room at thermal comfort until 41 days of age, when 90 animals (30 from each diet) were subjected to acute heat stress of 38°C for 24 hours. After 24 hours of stress, the animals from both groups (comfort and heat stress, HS) were slaughtered via cervical dislocation at 42 days.

### Body temperature and plasma analysis

Before slaughtering, the rectal temperature was measured in the thermal comfort and HS birds from both the starter and grower evaluated periods. Blood from five animals was collected for creatinine content analysis. The blood was collected from the jugular veins into heparin tubes and was stored on ice. After centrifugation (3.024 × g, 10 min, 4°C), the plasma was collected and stored at -20°C until analysis. Creatinine analyses were performed according to colorimetric methods with the kit, Creatinine-PP MS80022230066, following the manufacturer’s recommendations (Gold Analisa, Belo Horizonte, Minas Gerais, Brazil).

### Gene expression

For gene expression analysis, samples of liver and breast muscle (*Pectoralis superficialis*) were collected from five animals from each treatment for the starter and grower periods, and stored in RNA Holders (BioAgency Biotecnologia, São Paulo, Brasil) at -20°C until total RNA extraction.

Total RNA was extracted using Trizol (Invitrogen, Carlsbad CA, USA) according to the manufacturer’s instructions (1 mL per 100 mg of tissue). All materials used were previously treated with the RNase inhibitor, RNase AWAY (Invitrogen, Carlsbad, CA, USA). The tissue and Trizol mixture was triturated with a Polytron electric homogenizer until completely dissociated. Next, 200 μL chloroform was added to the sample, and the mixture was manually homogenized for 1 minute. The samples were then centrifuged for 15 minutes at 12,000 rpm and 4°C. The aqueous phase was collected and transferred to a clean tube containing 500 μL isopropanol per tube and again homogenized and centrifuged for 15 minutes at 12,000 rpm and 4°C. The supernatant was discarded, and the precipitate was washed in 1 mL 75% ethanol. The material was centrifuged again at 12,000 rpm for 5 minutes, and the supernatant was discarded. The pellet was dried for 15 minutes and resuspended in ultrapure RNase-free water.

The total RNA concentration was measured using a spectrophotometer at a wavelength of 260 nm. The RNA integrity was analyzed using a 1% agarose gel stained with 10% ethidium bromide and visualized under ultraviolet light. The RNA samples were treated with DNase I (Invitrogen, Carlsbad, CA, USA) according to the manufacturer’s instructions to remove possible genomic DNA contamination.

A SuperScript III First-Strand Synthesis Super Mix (Invitrogen, Carlsbad, CA, USA) kit was used for cDNA synthesis according to the manufacturer’s instructions. For this reaction, 6 μL of total RNA, 1 μL of oligo dT (50 μM oligo(dT)_20_) and 1 μL of annealing buffer were added to a sterile RNA-free tube. The reaction was then incubated for 5 minutes at 65°C and placed on ice for 1 minute. Subsequently, 10 μL of 2× First-Strand Reaction Mix and 2 μL of solution containing SuperScript III reverse transcriptase enzyme and RNase inhibitor were added to the tubes. The solution was incubated for 50 minutes at 50°C for the synthesis of complementary DNA. Next, the reaction was incubated for 5 minutes at 85°C and immediately placed on ice. The samples were stored at -20°C until use.

Real-time PCR reactions were performed using the fluorescent dye SYBR GREEN (SYBR GREEN PCR Master Mix, Applied Biosystems, Carlsbad, CA). All reactions were analyzed under the same conditions and normalized to the ROX Reference Dye (Invitrogen, Carlsbad, CA, USA) to correct for fluctuations in the readings due to evaporation during the reaction.

The primers used in the IGF-I, GHR, PIK3R1, atrogin-1, and CTSL2 amplification reactions were designed based on the gene sequences deposited at www.ncbi.nlm.nih.gov (accession numbers: FJ977570.1, NM001001293.1, XM_424759.3, NM_001030956, NM_001168009.1, respectively) using the site www.idtdna.com (Table 2). IGF-I, GHR, and PI3KR1 gene expression was evaluated in the liver. Atrogin-1 and CTSL2 gene expression was evaluated in the muscle. Two endogenous controls, ß-actin and GAPDH, were tested, and ß-actin (accession number L08165) was selected because its amplification was more efficient and ß-actin had no variation across treatments. All trials were performed in a final volume of 25 μL and in duplicate.

**Table 2:** Primer sequences used for quantitative real-time polymerase chain reaction.

Gene	Amplicon^1^	Temperature (ºC)^2^	Primers sequence (5’- 3’)
GHR^3^	145	60°C	AACACAGATACCCAACAGCC
			AGAAGTCAGTGTTTGTCAGGG
IGF-I	140	60°C	CACCTAAATCTGCACGCT
			CTTGTGGATGGCATGATCT
PI3KR1	145	60°C	GCCCTCTCCTTTTCAAAT
			ACAGTATTAGGTTTCGGTGGC
CTSL2	80	60°C	GAAGTCAGAAAGGAAGTACAGAGG
			CTCTCCAGTCAACAGATCGTG
Atrogin-1	174	60°C	CCAACAACCCAGAGACCTGT
			GGAGCTTCACACGAACATGA
β- actin	136	60°C	ACCCCAAAGCCAACAGA
			CCAGAGTCCATCACAATACC

^1^Amplicon (bp)

^2^Annealing Temperature (°C)

^3^CTSL2, cathepsin L2; GHR, growth hormone receptor; IGF-I, insulin-like growth factor I; PI3KR1, phosphatidylinositol 3-kinase, regulatory 1.

The primers for the analyzed genes were adequate for real time PCR analysis. The amplification efficiency was similar for the genes of interest, with 90 to 110% efficiency. The analysis of the dissociation curves did not reveal any presence of unspecific products or the formation of primer dimers, demonstrating the reliability of the data in the estimated mRNA expression of the evaluated genes. The β-actin used as an endogenous control did not show any statistically significant difference across the treatments. This verified the efficiency of its use as the endogenous control.

### Statistical analysis

Statistical analysis was performed separately for each period. The 2^-ΔCT^ method was used to analyze the relative expression [9]. The data were analyzed using the GLM procedure, and the means were compared using Tukey’s test (P < 0.05) (SAS Inst. Inc., Cary, NC, USA). The results are expressed as means and standard error.

## Results

**Fig 1:**
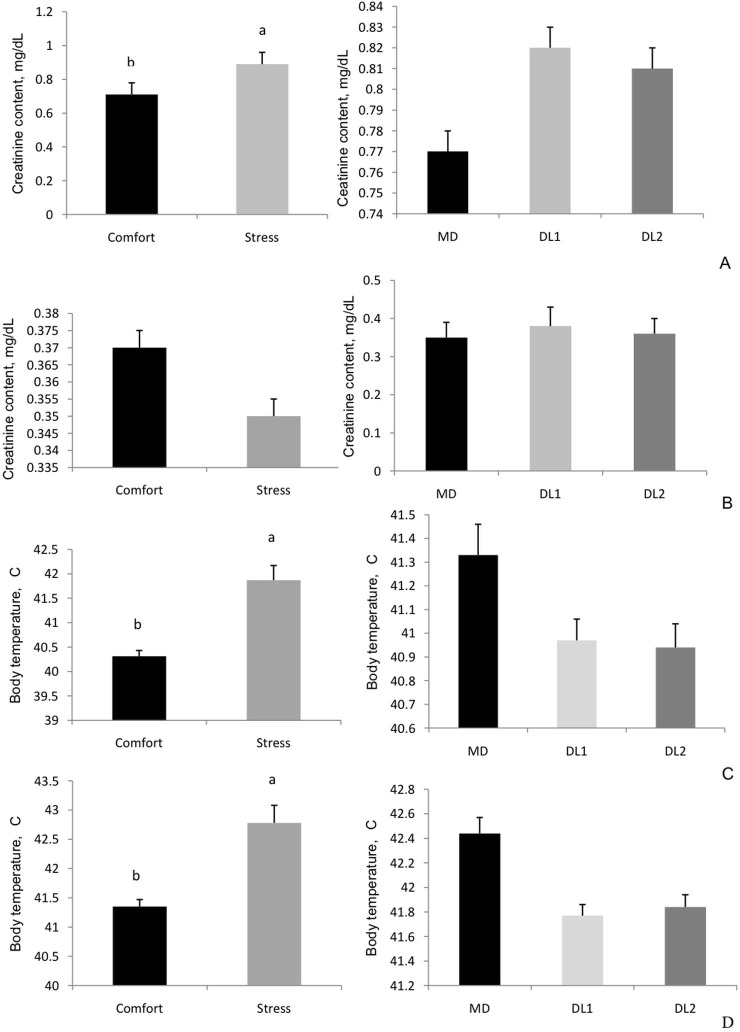
Effects of environment and methionine supplementation on creatinine content and body temperature of broilers from the starter (A and C) and grower (B and D) periods.

No effect of the interaction between methionine supplementation and temperature was observed on body temperature or creatinine content (Fig. 1). We observed that acute heat stress (38°C for 24 hours) was sufficient to increase the birds' body temperature in both the experiments, with 40.31°C ± 0.07 (thermal comfort) *vs*. 41.87°C ± 0.17 (HS) (P<0.0001) for animals in the starter period and 41.35°C ± 0.19 (thermal comfort) *vs*. 42.78°C ± 0.12 (HS) (P<0.0001) for animals in the grower phase. HS animals from the starter phase also presented higher creatinine content (P<0.0001).

The results of the IGF-I, GHR, PI3KR1, CTSL2, and atrogin-1 gene expression of the broilers at 1–21 days of age are shown in Table 3. In this phase, no effect of the interaction between methionine supplementation and temperature was observed on gene expression.

**Table 3:** IGF-I, GHR, PI3KR1, CTSL2, and atrogin 1 gene expression of broilers from the starter period.

		IGFI-I^2^		GHR		PI3KR1		CTSL2		Atrogin1^3^	
		Mean	SE	Mean	SE	Mean	SE	Mean	SE	Mean	SE
Comfort	MD^1^	0.90	0.07	1.89	0.21	0.036	0.00	0.019	0.001	1.56	0.34
	DL1	1.14	0.15	3.33	0.62	0.059	0.02	0.012	0.001	1.21	0.24
	DL2	0.90	0.27	4.95	1.17	0.051	0.01	0.014	0.001	0.63	0.16
Stress	MD	0.47	0.14	1.84	0.66	0.050	0.00	0.050	0.008	1.61	0.43
	DL1	1.06	0.26	2.53	0.73	0.054	0.00	0.027	0.004	1.37	0.25
	DL2	0.88	0.13	4.70	1.87	0.039	0.00	0.031	0.002	0.92	0.14
Main effects											
Environment	Comfort	0.98	0.18	3.39	1.79	0.049	0.02	0.015^b^	0.007	1.14	0.57
	Stress	0.80	0.23	3.02	2.34	0.048	0.03	0.036^a^	0.013	1.30	0.56
Diet	MD	0.68^b^	0.17	1.87^b^	0.47	0.043	0.02	0.035^a^	0.011	1.59^a^	0.37
	DL1	1.10^a^	0.20	2.93^b^	0.69	0.057	0.01	0.019^b^	0.006	1.29^a^	0.24
	DL2	0.89^ab^	0.20	4.82^a^	1.49	0.045	0.01	0.023^b^	0.006	0.78^b^	0.17
Probabilities											
Environment		NS		NS		NS		<0.0001		NS	
Diet		0.0144		0.0011		NS		0.0004		0.0012	
Interaction		NS		NS		NS		NS		NS	

^a, b^ Mean values within a column with different superscript letters are significantly different (P<0.05).

^1^MD, methionine deficient; DL1, recommended level of methionine supplementation; DL2, methionine supplementation in excess.

^2^CTSL2, cathepsin L2; GHR, growth hormone receptor; IGF-I, insulin-like growth factor I; PI3KR1, phosphatidylinositol 3-kinase, regulatory 1.

^3^Expressed as arbitrary units (AU).

NS, non significant.

The HS animals had lower CTSL2 gene expression than the animals that remained in comfort conditions (0.015 *vs*. 0.036 arbitrary units, AU; P<0.0001). For diet, we observed an effect of methionine supplementation on IGF-I, GHR, CTSL2, and atrogin-1 gene expression. The animals on the DL1 diet presented higher IGF-I expression than the animals on the MD diet (P = 0.0144). The highest GHR expression and the lowest atrogin-1 expression were observed in the animals fed the DL2 diet (P = 0.0011). The animals on the MD diet presented the highest CTSL2 expression (P = 0.0004).

The results of the gene expression of the broilers at 22–42 days of age are shown in Table 4. Methionine supplementation and heat stress had no effects on IGF-I or PI3KR1 gene expression in the animals from the grower phase. Only methionine supplementation had an effect on atrogin-1 gene expression (P<0.0001), and higher methionine content in the diet caused lower atrogin-1 gene expression.

**Table 4:** IGF-I, GHR, PI3KR1, CTSL2, and atrogin 1 gene expression of broilers from the grower period.

		IGFI-I^2^		GHR		PI3KR1		CTSL2		Atrogin 1^3^	
		Mean		SE	Mean	SE	Mean	SE		Mean	SE	Mean	SE
Comfort	MD^1^	0.22	0.02	2.10^bc^	0.24	0.017	0.002	0.024^cd^	0.005	1.83	0.4
	DL1	0.28	0.04	3.69^b^	0.69	0.014	0.005	0.015^d^	0.003	1.42	0.2
	DL2	0.24	0.06	5.49^a^	1.29	0.014	0.002	0.013^d^	0.002	0.74	0.2
Stress	MD	0.14	0.07	2.04^c^	0.74	0.012	0.001	0.074^a^	0.010	2.34	0.5
	DL1	0.26	0.06	2.80^bc^	0.80	0.013	0.002	0.034^bc^	0.005	1.61	0.3
	DL2	0.22	0.03	2.26^bc^	0.67	0.02	0.006	0.038^b^	0.004	1.09	0.1
Main effects											
Environment	Comfort	0.25	0.04	3.76	1.15	0.015	0.003	0.017	0.004	1.33	0.4
	Stress	0.21	0.06	2.37	0.72	0.015	0.006	0.049	0.012	1.68	0.4
Diet	MD	0.18	0.05	2.06	0.53	0.015	0.005	0.049	0.017	2.08^a^	0.4
	DL1	0.27	0.05	3.25	0.77	0.013	0.004	0.024	0.007	1.51^b^	0.2
	DL2	0.23	0.05	3.88	1.38	0.017	0.005	0.026	0.008	0.92^c^	0.2
Probabilities											
Environment		NS		0.0053		NS		<0.0001		NS	
Diet		NS		0.0113		NS		<0.0001		<0.0001	
Interaction		NS		0.0252		NS		0.0011		NS	

^a, b, c, d^ Mean values within a column with different superscript letters are significantly different (P<0.05).

^1^MD, methionine deficient; DL1, recommended level of methionine supplementation; DL2, methionine supplementation in excess.

^2^CTSL2, cathepsin L2; GHR, growth hormone receptor; IGF-I, insulin-like growth factor I; PI3KR1, phosphatidylinositol 3-kinase, regulatory 1.

^3^Expressed as arbitrary units (AU).

NS, non significant.

There was an interaction (methionine vs. HS) for GHR (P = 0.0252) and CTSL2 (P = 0.0011) gene expression. The highest GHR expression was observed in the animals maintained in thermal comfort on the DL2 diet, whereas the lowest expression was in the HS birds fed the MD diet. The highest CTSL2 gene expression was observed in the broilers under HS and fed the MD diet. The animals housed at thermal comfort on the DL1 and DL2 diets presented the lowest CTSL2 expression.

## Discussion

It has been shown that high temperatures are associated with elevated body temperature of birds. In this study, it was observed that acute (24 hours) heat stress increased the body temperature of the birds even in the starter period (1–21 days old). The higher body temperature observed in the HS animals induced metabolic changes, such as increased ROS production, increased lipid peroxidation [10], decreased bird performance and damage to the parts yield [11,12]. References in the literature show that broilers under high temperatures increase their plasma corticosterone levels, which stimulates protein turnover in broilers, increasing in particularly the protein breakdown in the body [12]. Because the flux blood in the organs is reduced to stimulate increased amounts of blood in the peripheral tissues, which leads to a loss of body heat to the environment and lower relative weights of many organs, such as the intestine, liver, gizzard, lungs, proventriculus, and heart [13]. There is also a reduction in the absorptive area of the intestine [14], as well as a reduction in parts yield [11] and an increase in water consumption [13]. These physiological changes indicate that broilers are reducing their protein deposition.

Our study is consistent with data presented in other studies because we observed that animals maintained under heat stress presented a higher plasma creatinine concentration. The plasma concentration of creatinine is normally associated with renal functions; however, some studies have shown that high levels of creatinine are present in the blood when there is a greater breakdown or turnover of protein and that renal deficiency is possibly associated with the high turnover rates [15].

In our study, we assessed the expression of IGF-I, GHR, and PI3KR1 genes in the liver because a study performed by our research group showed that the liver is the primary site of IGF-I production [2]. Expression of the CTSL2 and atrogin-1 genes was performed in the muscle because results from the literature indicate the importance of the ubiquitin-proteasome pathway [4–5] and the role of lysosomal degradation in muscular atrophy [7].

Decreased protein synthesis capacity and rate, lower growth rate, lower efficiency of protein deposition, and lower RNA levels have also been observed in animals exposed to heat stress [16]. Chickens exposed to high temperatures presented lower circulating IGF-I concentrations, along with lower T3 and T4 levels, higher corticosterone levels, higher TBARS levels, and greater antioxidant activity [17]. IGF-I activity is important not only for promoting protein synthesis but also for decreasing the protein degradation rate in the ubiquitin-proteosome complex [1].

Hormonal growth regulation involves a complex series of interactions between different hormones, with the somatotropic axis (GH, GHR, and IGF-I) considered to be the most important. GH can directly affect growth, but its effects are primarily mediated through IGF-I activity. The presence of GH in an organism promotes the synthesis and release of this hormone [18]. The effect of GH on IGF-I is mediated by the GH receptor (GHR) because GH-GHR binding is necessary to stimulate IGF-I synthesis and release.

The binding between IGF-I and its receptor results in auto-phosphorylation and conformational changes, which produce a signaling cascade involving many proteins. Among these is the insulin receptor substrate (IRS), which is also phosphorylated by the IGF-I receptor, phosphatidylinositol 3-kinase (PI3K), phosphoinositide-dependent kinase 1 (PDK1), and PKB/Akt. After these last two proteins are recruited, the phosphorylation/activation of PKB/Akt by PDK1 occurs. Akt activation is important because this protein has a positive effect on protein synthesis. Akt activation stimulates mTOR, which controls a large number of components related to initiation and elongation [19]. However, Akt activation does have a negative effect on protein degradation because it acts directly on FoxO family complexes [20]. Akt phosphorylation inhibits the transcription factors of the FoxO family. The FoxO family is necessary to activate the MURF-1 and antrogin-1 enzymes [21] that act on the degradation pathway of the ubiquitin proteasome.

The degradation pathway of the ubiquitin proteasome consists of enzymatic actions that result in the release of amino acids after the breakdown of proteins linked to ubiquitin. The following three enzymes are necessary for this action: E1, a Ub-activating enzyme; E2, a Ub-carrier; and E3, a Ub-protein ligase. E3 is considered the key enzyme because it is responsible for recognizing the protein that will be the substrate of degradation and for ubiquitin transfer [22]. The MURF-1 and atrogin-1 enzymes are among the E3s, and because of their role in the degradation process, the coding gene of theses enzymes are called atrogenes and their expression is high in stressful situations [5] and during the deprivation of energy or amino acids [4, 22].

In our study, we observed that a proper increase of methionine levels to reach the broiler requirement by supplementing diets with DL-methionine was beneficial and did not induce any damage to the bird. Furthermore, higher amounts of methionine were associated with greater GHR and IGF-I gene expression, and although we did not observe variation in PI3KR1 gene expression, supplementation with the higher methionine level yielded decreased atrogin-1 gene expression. Perhaps, in addition to stimulating protein synthesis through a positive effect on IGF-I gene expression, methionine supplementation may also stimulate proteins other than PI3KR1 and upstream of atrogin-1, thereby signaling less degradation. Because of the complexity and numerous factors involved in the sensitive relationship between protein synthesis and degradation, various studies have been performed to evaluate environmental and dietary nutrient effects [17, 23] on the expression of genes involved in this metabolism. Several studies demonstrated that in addition to a greater effect on expression and IGF-I circulation [24, 25], there is a direct action of amino acids on the protein complex with mTOR. These studies suggest that the metabolic pathway by which amino acids act is distinct from that of the hormones and occurs not via PI3K but via Vps34 [26, 27].

Protein degradation can occur through enzymes present in lysosomes, and regardless of the form in which the substrate is brought into the interior of the lysosomes, they will be denatured by the low lysosomal pH and degraded by the same lysosomal proteases. The cathepsins B, D, and K and the lysosomal cysteine protease cathepsin L are among these proteins [28]. For the ubiquitin-proteasome pathway, studies also show the effects of nutrient deficiency and stress on this degradation pathway. The lack of amino acids induces autophagy not only by decrease stimulation of mTOR but also because of the formation of complexes essential for the formation of the autophagosome [29, 30]. Moreover, in our study, we found that a deficiency in methionine led to increased expression of the gene CTSL2.

The expression of cathepsin L2 was also greater in animals under heat stress than in animals under thermal comfort conditions. Just as the lack of amino acids and other nutrients affects proteolysis, stress is also responsible for initiating actions that promote proteolysis. This is because, apart from the effects of increased ROS production on metabolism, stress is also associated with a lower expression of IGF-I, an increased expression of components of the ubiquitin-proteasome pathway [5], activation of FoxO signaling [6], and induction of autophagy [7], similar to that observed in our study.

These results suggest that acute heat stress signals increased protein degradation because a higher expression of the cathepsin L2 gene occurred in stressed broilers. Furthermore, methionine supplementation may stimulate protein deposition, thereby ensuring not only higher expression of genes related to synthesis but also lower expression of genes related to degradation. Additional studies should be performed with different species to confirm our results.
